# Clinical Utility of 18F-FDG PET/CT in the Assessment of Mantle Cell Lymphoma With Intraorbital Involvement: A Case Report and Literature Review

**DOI:** 10.7759/cureus.105929

**Published:** 2026-03-26

**Authors:** David Gutierrez Albenda, Akira Osawa Pivovarov, Valeria Armani Arce, Álvaro Montoya Porras, Ian Taylor Roldán

**Affiliations:** 1 Cyclotron-PET/CT Laboratory, Universidad de Costa Rica, San José, CRI; 2 School of Medicine, Universidad de Costa Rica, San José, CRI

**Keywords:** 18f-fluorodeoxyglucose positron emission tomography (18f-fdg pet), mantle cell, mantle cell lymphoma, orbital lymphoma, pet ct scan

## Abstract

Mantle cell lymphoma (MCL) is an aggressive subtype of non-Hodgkin lymphoma with heterogeneous clinical presentations in which orbital involvement is uncommon. We present the case of a 66-year-old woman with progressive facial swelling, disseminated cutaneous lesions, and blurred vision in the left eye. A skin biopsy confirmed the diagnosis of MCL. Contrast-enhanced computed tomography demonstrated cervical lymphadenopathy and periocular masses. A fluorine-18 fluorodeoxyglucose positron emission tomography/computed tomography (18F-FDG PET/CT) study revealed increased metabolic uptake in an intraconal lesion within the left orbit in close relationship with the globe and the optic nerve, as well as nodal and extranodal involvement in multiple regions, suggesting intraorbital extranodal extension of MCL. This case highlights the potential value of 18F-FDG PET/CT in identifying intraorbital involvement and providing additional information for staging and therapeutic planning. Evidence regarding the role of 18F-FDG PET/CT in orbital manifestations of MCL remains limited, and additional studies are needed to better define its clinical utility in this setting.

## Introduction

Non-Hodgkin lymphomas (NHL) represent a heterogeneous group of malignancies characterized by different clinical courses and treatment responses even within the same class of lymphoma. Among them, mantle cell lymphoma (MCL) is considered an aggressive subtype, accounting for approximately 3-10% of NHL cases in Western countries, with a reported five-year overall survival ranging from 35% to 81% depending on different prognostic factors [1].

In ophthalmology, lymphomas involving ocular structures are generally classified into intraocular lymphoma and ocular adnexal lymphoma depending on the anatomical structures affected [2]. Orbital involvement by MCL is rare, and the available literature mainly consists of isolated case reports.

Staging of MCL requires integration of clinical findings, biomolecular markers, and imaging studies in order to determine disease extent and guide therapeutic decision-making. Computed tomography has traditionally been used for staging purposes; however, more recent evidence supports the role of fluorine-18 fluorodeoxyglucose positron emission tomography/computed tomography (18F-FDG PET/CT) for staging and response assessment in lymphoma [3].

In this report, we present the case of a 66-year-old woman with an insidious clinical presentation characterized by progressive facial swelling and disseminated cutaneous lesions, associated with visual disturbance in the left eye manifested as blurred vision. Histopathological examination of a skin lesion confirmed the diagnosis of MCL. Subsequent 18F-FDG PET/CT imaging demonstrated systemic lymphoproliferative disease, including intraorbital involvement, illustrating the potential utility of this imaging modality in identifying uncommon extranodal manifestations and supporting the staging of MCL.

## Case presentation

A 66-year-old woman with no significant past medical history presented with progressive facial swelling and the development of multiple skin lesions distributed across different body regions. In addition, the patient reported visual disturbance in the left eye manifested as blurred vision. Initially, she attributed this symptom to a possible change in her optical prescription due to her regular use of corrective lenses and did not seek immediate medical attention.

Due to the progression of the cutaneous lesions, a biopsy of one of the lesions was performed. Histopathological and immunophenotypic analysis confirmed the diagnosis of MCL.

Following the diagnosis, a contrast-enhanced CT scan of the head and neck was performed, revealing multiple cervical lymphadenopathies and several soft-tissue lesions involving the infratemporal region, facial soft tissues, and periorbital structures, including a mass in close contact with the left ocular globe suggestive of orbital involvement.

Given these findings suggestive of lymphomatous infiltration, further evaluation with 18F-FDG PET/CT imaging was requested.

A high-resolution PET acquisition was performed using a Biograph Vision 600 PET/CT system (Siemens Healthineers, Erlangen, Germany) equipped with lutetium-yttrium oxyorthosilicate (LYSO) detectors, with axial plane acquisitions at a speed of 1 mm/s. This was combined with a low-dose CT scan (CareDose, Nagpur, India) using a 128-slice multidetector helical scanner with 5-mm slices and a pitch of 0.6. The CT component was performed without intravenous contrast. The radiopharmaceutical 18F-FDG was administered intravenously through the left upper limb at a dose of 10.4 mCi.

**Figure 1:**
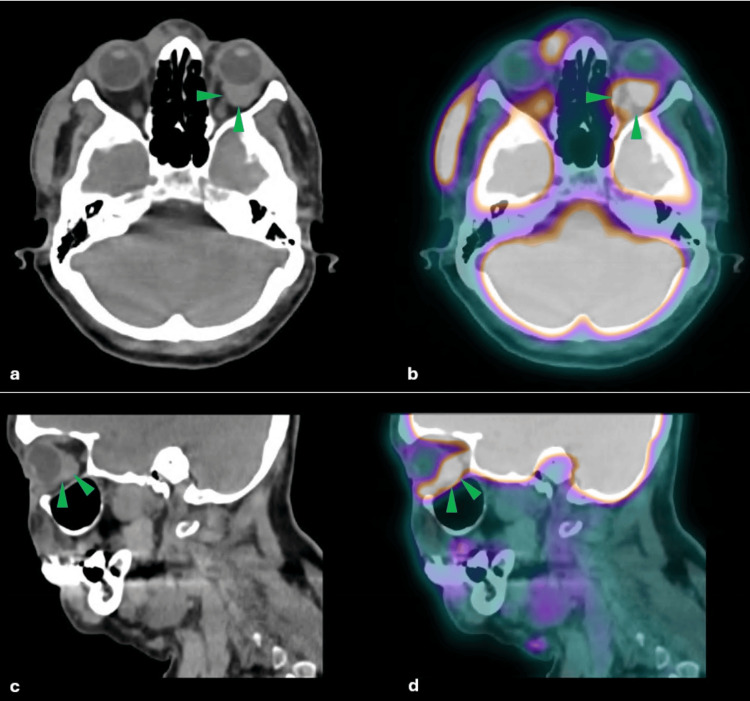
(a) Axial CT and (b) axial 18F-FDG PET/CT images demonstrating a hypermetabolic soft-tissue lesion within the left intraconal space (green arrowhead) in close contact with the posteroinferior aspect of the globe and partially encasing the optic nerve. (c) Sagittal CT and (d) sagittal 18F-FDG PET/CT images confirming the same lesion, measuring approximately 2.1 cm. 18F-FDG PET/CT: fluorine-18 fluorodeoxyglucose positron emission tomography/computed tomography.

**Figure 2:**
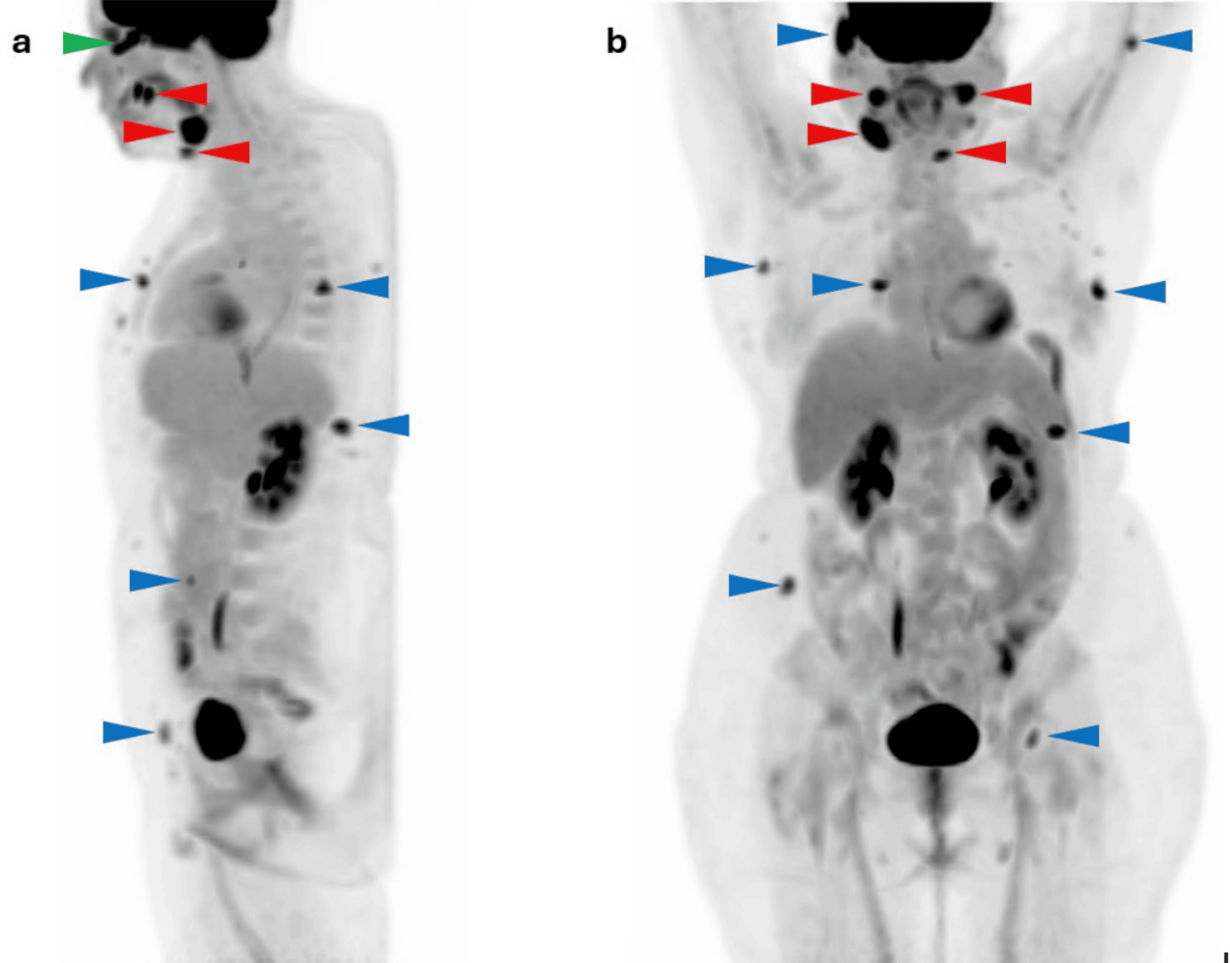
(a, b) MIP images from 18F-FDG PET/CT showing a hypermetabolic mass in the left intraconal space (green arrowhead); hypermetabolic cervical lymph nodes are seen (red arrowheads), along with multiple subcutaneous hypermetabolic foci corresponding to cutaneous lesions (blue arrowheads), consistent with disseminated lymphoproliferative disease with nodal and extranodal involvement. MIP: maximum intensity projection; 18F-FDG PET/CT: fluorine-18 fluorodeoxyglucose positron emission tomography/computed tomography.

The 18F-FDG PET/CT study demonstrated a hypermetabolic soft-tissue lesion within the left intraconal space (Figure 1a-d) in close contact with the posteroinferior aspect of the globe and partially encasing the optic nerve, measuring approximately 2.1 cm. Additional hypermetabolic lymphadenopathy was observed in the cervical lymph nodes involving levels Ia, Ib, IIa, IIb, III, and V, as well as in a right intraparotid lymph node measuring 2.7 cm (Figure 2a and b). Multiple hypermetabolic foci were also identified in the subcutaneous tissues of the head, neck, trunk, and proximal segments of the upper and lower extremities, corresponding to the clinically observed cutaneous lesions (Figure 2a and b). Overall, the PET/CT findings were consistent with disseminated lymphoproliferative disease with nodal and extranodal involvement, including intraorbital extension.

Orbital biopsy was not performed due to the previously confirmed diagnosis of MCL from the skin biopsy and the presence of systemic disease.

## Discussion

Current epidemiology of orbital lymphomas

Current evidence suggests that orbital lymphomas account for around 50-60% of ocular adnexal lymphomas [4]; however, prevalence tends to differ depending on the population studied. An epidemiological study by Alfaar et al. [5] sought to examine the epidemiological profile of orbital lymphomas in the United States. Some of their findings include an age-standardized incidence rate (ASIR) of 2.6 persons per million (ppm), higher incidence rates in the orbit (ASIR = 1.24 ppm), NHL being the most prevalent subtype (85.4%), and a marked racial disparity: Asia-Pacific Islanders showed an ASIR of 1.3 ppm. Hsu et al. [6] performed a similar epidemiological review in Taiwan, finding MALT lymphomas as the main subtype in their studied population (67.9%), and the orbit as the most frequent anatomical site (39.3%). Certain countries, such as South Korea and Canada, have also noted an increased incidence in the number of cases of orbital lymphoma [7,8]. 

These epidemiological studies have also yielded some information as to some characteristics of these neoplasms regarding clinical presentation and histological subtype. Older adults are more frequently afflicted by orbital lymphomas, with a median age at diagnosis that ranges from the sixth to seventh decades of life [6-8]. These neoplasms tend to present both unilaterally and in an early stage [4]. Across multiple studies, it is evident that B-cell lymphomas, specifically extranodal marginal zone B-cell lymphomas, are the most prevalent histological subtypes; these account for 45-67% of cases [4-8]. 

Most available epidemiological data originate from North America and Asia, while epidemiological studies from Latin American countries remain limited. Some authors have suggested that the higher prevalence of lymphotropic viruses such as Epstein-Barr virus in Latin American populations may influence the distribution of lymphoma subtypes and age at presentation [9].

Role of 18F-FDG PET/CT for diagnosis of MCL

Current international guidelines recommend the use of 18F-FDG PET/CT as an initial staging imaging study of lymphomas, including MCL [10]. Certain imaging visual characteristics, like increased focal uptake in nodal and extranodal sites and distinguishing it from physiological uptake, are the main values of 18F-FDG PET/CT. Yet there is no literature regarding the sensitivity and specificity of the exact role of this imaging modality in extranodal MCL, nevertheless, intraorbital MCL. 

An Italian multicentric study by Albano et al. [11] concluded that MCL is an FDG-avid lymphoma and so 18F-FDG PET/CT can be a useful tool for staging purposes, making it a considerable diagnostic flow-chart for patients with MCL. The study showed good specificity for bone marrow and gastrointestinal tract evaluations, yet orbital involvement was not documented in the 229 patients enrolled in the study. 

Another large single-center retrospective review by Zanni et al. [12] evaluated the role of 18F-FDG PET/CT in the diagnosis of ophthalmologic lymphoma as the primary site and concluded that the value of 18F-FDG PET/CT is highly dependent on the histologic subtype of lymphoma, with MCL having almost 100% uptake. Yet only 68% of ocular adnexal lymphoma had positive FDG uptake, concluding that this imaging modality does not appear to be useful for the diagnosis of ocular adnexal lesions. 

In the present case, intraorbital involvement occurred as a manifestation of systemic MCL rather than as a primary ocular adnexal lymphoma. Therefore, the high metabolic activity characteristic of MCL facilitated the detection of the orbital lesion on PET/CT. These findings suggest that PET/CT may be particularly useful for identifying uncommon extranodal manifestations of systemic lymphoma.

## Conclusions

18F-FDG PET/CT plays an important role in the staging of MCL due to its ability to detect metabolically active nodal and extranodal disease. In the present case, PET/CT allowed identification of intraorbital involvement within the intraconal space in the context of disseminated disease, providing additional information beyond conventional CT imaging and contributing to a more comprehensive assessment of disease extent. Orbital involvement in MCL is uncommon and has been described only rarely in the literature. Furthermore, the intraorbital lesion in our patient occurred as part of systemic MCL with multiple extranodal manifestations, including cutaneous involvement.

This case highlights the potential utility of PET/CT in detecting rare extranodal sites of disease and illustrates its value in the evaluation of atypical clinical presentations of MCL. Additional case series and clinical studies are needed to further define the role of PET/CT in orbital manifestations of MCL.
